# Soy intake and breast cancer risk: a prospective study of 300,000 Chinese women and a dose–response meta-analysis

**DOI:** 10.1007/s10654-019-00585-4

**Published:** 2019-11-21

**Authors:** Yuxia Wei, Jun Lv, Yu Guo, Zheng Bian, Meng Gao, Huaidong Du, Ling Yang, Yiping Chen, Xi Zhang, Tao Wang, Junshi Chen, Zhengming Chen, Canqing Yu, Dezheng Huo, Liming Li, the China Kadoorie Biobank Collaborative Group, Junshi Chen, Zhengming Chen (PI), Robert Clarke, Rory Collins, Yu Guo, Liming Li (PI), Jun Lv, Richard Peto, Robin Walters, Daniel Avery, Ruth Boxall, Derrick Bennett, Yumei Chang, Yiping Chen, Zhengming Chen, Robert Clarke, Huaidong Du, Simon Gilbert, Alex Hacker, Mike Hill, Michael Holmes, Andri Iona, Christiana Kartsonaki, Rene Kerosi, Ling Kong, Om Kurmi, Garry Lancaster, Sarah Lewington, Kuang Lin, John McDonnell, Iona Millwood, Qunhua Nie, Jayakrishnan Radhakrishnan, Paul Ryder, Sam Sansome, Dan Schmidt, Paul Sherliker, Rajani Sohoni, Becky Stevens, Iain Turnbull, Robin Walters, Jenny Wang, Lin Wang, Neil Wright, Ling Yang, Xiaoming Yang, Zheng Bian, Yu Guo, Xiao Han, Can Hou, Jun Lv, Pei Pei, Chao Liu, Yunlong Tan, Canqing Yu, Zengchang Pang, Ruqin Gao, Shanpeng Li, Shaojie Wang, Yongmei Liu, Ranran Du, Yajing Zang, Liang Cheng, Xiaocao Tian, Hua Zhang, Yaoming Zhai, Feng Ning, Xiaohui Sun, Feifei Li, Silu Lv, Junzheng Wang, Wei Hou, Mingyuan Zeng, Ge Jiang, Xue Zhou, Liqiu Yang, Hui He, Bo Yu, Yanjie Li, Qinai Xu, Quan Kang, Ziyan Guo, Dan Wang, Ximin Hu, Jinyan Chen, Yan Fu, Zhenwang Fu, Xiaohuan Wang, Min Weng, Zhendong Guo, Shukuan Wu, Yilei Li, Huimei Li, Zhifang Fu, Ming Wu, Yonglin Zhou, Jinyi Zhou, Ran Tao, Jie Yang, Jian Su, Fang liu, Jun Zhang, Yihe Hu, Yan Lu, Liangcai Ma, Aiyu Tang, Shuo Zhang, Jianrong Jin, Jingchao Liu, Zhenzhu Tang, Naying Chen, Ying Huang, Mingqiang Li, Jinhuai Meng, Rong Pan, Qilian Jiang, Jian Lan, Yun Liu, Liuping Wei, Liyuan Zhou, Ningyu Chen, Ping Wang, Fanwen Meng, Yulu Qin, Sisi Wang, Xianping Wu, Ningmei Zhang, Xiaofang Chen, Weiwei Zhou, Guojin Luo, Jianguo Li, Xiaofang Chen, Xunfu Zhong, Jiaqiu Liu, Qiang Sun, Pengfei Ge, Xiaolan Ren, Caixia Dong, Hui Zhang, Enke Mao, Xiaoping Wang, Tao Wang, Xi zhang, Ding Zhang, Gang Zhou, Shixian Feng, Liang Chang, Lei Fan, Yulian Gao, Tianyou He, Huarong Sun, Pan He, Chen Hu, Xukui Zhang, Huifang Wu, Pan He, Min Yu, Ruying Hu, Hao Wang, Yijian Qian, Chunmei Wang, Kaixu Xie, Lingli Chen, Yidan Zhang, Dongxia Pan, Qijun Gu, Yuelong Huang, Biyun Chen, Li Yin, Huilin Liu, Zhongxi Fu, Qiaohua Xu, Xin Xu, Hao Zhang, Huajun Long, Xianzhi Li, Libo Zhang, Zhe Qiu

**Affiliations:** 1 Department of Epidemiology and Biostatistics, School of Public Health, Peking University Health Science Center, 38 Xueyuan Road, Beijing, 100191 China; 2 Chinese Academy of Medical Sciences, Beijing, China; 3 Medical Research Council Population Health Research Unit, University of Oxford, Oxford, UK; 4 Clinical Trial Service Unit and Epidemiological Studies Unit (CTSU), Nuffield Department of Population Health, University of Oxford, Oxford, UK; 5 NCDs Prevention and Control Department, Maiji CDC, Tianshui, Gansu China; 6 China National Center for Food Safety Risk Assessment, Beijing, China; 7 Department of Public Health Sciences, The University of Chicago, 5841 S. Maryland Ave., MC2000, Chicago, IL 60637 USA; 8 Key Laboratory of Molecular Cardiovascular Sciences (Peking University), Ministry of Education, Beijing, China; 9 Peking University Institute of Environmental Medicine, Beijing, China

**Keywords:** Soy intake, Breast cancer, Prospective cohort study, Dose–response meta-analysis

## Abstract

Epidemiological evidence on the association of soy intake with breast cancer risk is still inconsistent due to different soy intake levels across previous studies and small number of breast cancer cases. We aimed to investigate this issue by analyzing data from the China Kadoorie Biobank (CKB) study and conducting a dose–response meta-analysis to integrate existing evidence. The CKB study included over 300,000 women aged 30–79 from 10 regions across China enrolled between 2004 and 2008, and followed-up for breast cancer events until 31 December 2016. Information on soy intake was collected from baseline, two resurveys and twelve 24-h dietary recalls. We also searched for relevant prospective cohort studies to do a dose–response meta-analysis. The mean (SD) soy intake was 9.4 (5.4) mg/day soy isoflavones among CKB women. During 10 years of follow-up, 2289 women developed breast cancers. The multivariable-adjusted relative risk was 1.00 (95% confidence interval [CI] 0.81–1.22) for the fourth (19.1 mg/day) versus the first (4.5 mg/day) soy isoflavone intake quartile. Meta-analysis of prospective studies found that each 10 mg/day increment in soy isoflavone intake was associated with a 3% (95% CI 1–5%) reduced risk of breast cancer. The CKB study demonstrated that moderate soy intake was not associated with breast cancer risk among Chinese women. Higher amount of soy intake might provide reasonable benefits for the prevention of breast cancer.

**Electronic supplementary material:**

The online version of this article (10.1007/s10654-019-00585-4) contains supplementary material, which is available to authorized users.

## Introduction

Breast cancer is the most common cancer and one of the leading causes of cancer death among women throughout the world [1]. Its incidence rate varies across regions, which is much higher in Western than in Asian countries [1–5]. This regional variation has been postulated to be attributed to the differences in lifestyle and dietary factors. One of such dietary components is soy foods, which are staples in Asian diet but rare in diet of western populations. Soy foods are major sources of dietary isoflavones, which share structural similarities with 17-β-estradiol and may serve as functional estrogen antagonists to protect against breast cancer [6, 7]. Also, soy isoflavones may exert effect through estrogen independent pathways [6].

Epidemiological evidence on the relationship between soy foods and breast cancer is still limited and inconclusive [8], despite potential mechanisms from experiments [6, 7]. Several meta-analyses concluded that higher amount of soy intake was associated with lower risk of incident breast cancer among Asian women but not among their Western counterparts [9–12]. However, between-study heterogeneity was obvious [9–12] and most original studies included in these meta-analyses were case–control studies [10–12]. By contrast, a recent meta-analysis involving only cohort studies, which are less subject to selection and recall bias, found no association between isoflavone intake and breast cancer, but it showed that a higher consumption of soy-based foods was weakly associated with a lower risk of breast cancer relative to a low consumption of soy foods (HR: 0.87, 95% CI 0.76–1.00) [13]. The definition of “high” soy food intake varies across studies in previous meta-analyses, making it difficult to interpret the pooled risk estimates. Meta-analysis taking into account the amount of soy foods or isoflavone dose is desirable.

Several cohort studies or nested case–control studies related to soy and incident breast cancer gave inconsistent results. Studies conducted in Western countries where dietary soy intake levels were low [14–24] or moderate [25, 26] have found no clear association. Evidence from studies in Asia where dietary soy intake levels were moderate to high was also inconsistent. Some studies found no statistically significant association [27, 28] while others reported a reduced breast cancer risk for women in the highest soy intake groups [29–32]. As most previous cohort studies in Asia have limited numbers of breast cancer cases, insufficient statistical power as well as different cut-off values for soy intake categories may explain this inconsistency. To our knowledge, only one cohort study, namely the Shanghai Women’s Health Study (SWHS) which enrolled participants from a highly developed city, was conducted in China previously [32]. More evidence from the general Chinese adults with diverse economic background is still lacking.

Using the data from the China Kadoorie Biobank (CKB), a large scale prospective cohort study involving over 300,000 women from 10 geographically and economically diverse regions in China, we evaluated the relationship between soy intake and risk of incident breast cancer. Subgroup analyses were also conducted to assess whether risk estimates varied by baseline characteristics like menopausal status. Participants’ soy consumption levels and cut-off values of soy categories varied across previous studies. In order to provide a clearer picture on the soy-breast cancer relationship, we also did a dose–response meta-analysis to integrate results of prospective cohort studies.

## Methods

### Study population

The CKB study recruited participants from 5 urban areas and 5 rural areas in China. The study design and methods have been described in detail elsewhere [33]. Briefly, between June 2004 and July 2008, the study enrolled 512,715 adults (302,510 women) aged 30–79 years who completed baseline data collection, including a questionnaire and physical measurements after signing a written informed consent form. Among the women recruited at baseline, we excluded persons with previously cancer diagnoses (*n* = 1610), with missing values for key variables (*n* = 47 for reproductive characteristics, n = 1 for body mass index [BMI]), leaving 300,852 female participants in the main analyses. Approval of the study was obtained from ethics committees or institutional review boards at the University of Oxford, the Chinese Center for Disease Control and Prevention, the Chinese Academy of Medical Sciences, and all participating centers.

### Assessment of soy consumption

In the baseline questionnaire, one item asked participants about the overall frequency of soy foods (e.g. soybeans, fresh tofu, fried tofu, pressed tofu, soymilk skin or film, soybean flakes, soymilk and so on) during the past 12 months: never/rarely, monthly, 1–3 days per week, 4–6 days per week, or daily. After participants completed the baseline survey, two resurveys were conducted in 2008 and 2013, respectively, each involving about 5% of the randomly selected participants from each of the 10 study regions. The first resurvey questionnaire asked exactly the same question on soy consumption as the baseline one, whereas the question was split into 2 items in the second resurvey. One item asked about the frequency and amount of soymilk consumption and the other item about soy foods other than soymilk. To evaluate the reproducibility and validity of food frequency questionnaires (FFQs) used in baseline and resurveys, an intensive dietary study of 432 CKB participants (254 women) was conducted from 2015 to 2016. These participants completed two FFQs (median interval: 3.3 months) and twelve 24-h dietary recalls (24-HDR). The 24-HDRs covered all kinds of soy foods in China and were conducted in three seasons separately, with 3 weekdays and 1 weekend day in each season. For female participants, the weighted Kappa statistic was 0.66 for reproducibility of baseline soy food frequency and 0.77 for reproducibility of soy consumption amount (excluding soy milk) in 2^nd^ resurvey. Using 24-HDRs as the gold standard, the weighted Kappa statistic was 0.67 for validity of baseline food frequency and 0.74 for validity of soy consumption amount (excluding soy milk) in 2^nd^ resurvey.

We combined intake frequency from baseline and 1st resurvey and the intake amount from the 2nd resurvey to estimate the usual amount of soy intake for each woman. Then, we converted the usual amount of soy intake into amount of soy isoflavone by taking into account the proportions that different kinds of soy foods contributing to total soy food amount among women in the 24-HDRs and the soy isoflavone content of different soy foods (Appendix).

### Assessment of covariates

Information on socio-demographic characteristics (age, education and household income), lifestyle factors (alcohol consumption, physical activity and dietary habits), reproductive characteristics (i.e. age at menarche, parity, breast-feeding duration, menopausal status, menopausal age, and use of oral contraceptives), and family history of cancer were obtained from the baseline questionnaire. Daily energy intake was calculated by taking into account the 12 groups of foods available in the present study. A participant was considered as having a family history of cancer if at least one of their parents or siblings was diagnosed with cancer. At baseline, body weight and height were measured by trained staff using calibrated instruments and BMI was calculated by weight (kg)/height squared (m^2^).

### Identification of breast cancer cases

Participants were followed-up from the date of completing baseline questionnaire to the date of diagnosis of breast cancer, death, loss to follow-up or 31 December 2016, whichever came first. By 31 December 2016, about 1% participants were censored due to loss to follow-up. Incident breast cancer cases were identified periodically through linkage with local disease and death registries [34], and the national health insurance system or ascertained through active follow up. All diseases were coded according to the International Classification of Diseases, 10th Revision (ICD-10), by trained staff blinded to baseline information of participants. Breast cancer was coded as C50. The proportion of death certificate only cases was 4.1% for breast cancer, indicating the high completeness of cancer registration in the present study.

### Statistical analysis

Baseline characteristics of participants were presented as means (standard deviations, SDs) or percentages across 4 categories of baseline soy consumption, standardized for age and study region if appropriate, via logistic regressions for categorical variables or multiple linear regressions for continuous variables.

Hazard ratios (HRs) and 95% confidence intervals (95% CIs) were calculated for breast cancer risk by both soy frequency and usual amount quartiles using Cox proportional hazard regression models. Categorical soy intake variables were treated as continuous variables to evaluate the linear trend. In the Cox models, age was used as the underlying time scale and baseline characteristics as covariates. Each Cox model was stratified by baseline age groups (in 5-year intervals) and study region. Potential confounding factors adjusted for in the multivariable models were: education attainment, household income, smoking status, alcohol consumption, physical activity, baseline BMI, standing height, age at menarche, parity, average breastfeeding duration, menopausal status and age at menopause, use of oral contraceptives, family history of cancer, total energy intake, and consumption frequency of fresh fruit, fresh vegetables, preserved vegetables, red meat, poultry, fish and dairy products at baseline. Information on hormone replacement therapy was not collected at baseline and thus was not adjusted for in the models. However, we believed it would not influence the risk estimates since < 1% of the females have ever used hormone replacement treatment according to data from the second resurvey. The method of Schoenfeld residuals found no violation of the proportional hazards assumption for soy intake in fully-adjusted model.

A stratified analysis was further performed separately among pre-menopausal and post-menopausal women to assess any modifying effect of menopause on the association of soy consumption and breast cancer. Besides, stratified analyses were conducted according to other baseline characteristics such as BMI. Multiplicative interactions were tested using likelihood ratio tests comparing models with and without the cross product terms between stratifying variables and soy food consumption categories.

Several sensitivity analyses were done separately: (1) by excluding females who developed breast cancer during the first 2 years of follow-up; or (2) by excluding those with a family history of cancer.

### Systematic review and dose–response meta-analysis

We searched on PubMed, Embase and Cochrane library from their dates of inception to March 2019 for prospective studies examining the association between soy intake and breast cancer. Studies using concentrations of isoflavones or their metabolites in biological samples were not included owing to the difficulty in converting biological concentration into amount of soy isoflavone intake. However, we believed that this had no great impact on the result because studies using biological concentrations as exposure assessment were mainly conducted among populations with pretty low level of soy intake [14, 16, 19]. We excluded studies if the amount of soy intake was unavailable and could not be estimated using relevant data. We also excluded reviews, non-human studies, abstract-only publications or editorials. If the same cohort study published more than one original articles on soy intake and breast cancer, the paper reporting the largest sample size, longest follow-up time, or the widest variation in soy intake levels was kept. In addition, studies in which participants’ soy intake levels were very low (i.e. mean or median intake < 5 mg/day of soy isoflavone for the highest consumption group) were not included in the quantitative synthesis (dose–response meta-analysis) as the weights contributed by these studies were negligible. Detailed literature searching strategy, data extraction methods, and quality assessment of individual study were presented in Appendix.

In the dose–response meta-analysis, HRs were considered as effect sizes and the median or mean soy isoflavone intake of each consumption category was regarded as the consumption dose of corresponding category. To test for potential non-linear relationship between soy isoflavone intake and incident breast cancer, a non-linear dose–response meta-analysis was done by coding soy isoflavone intake as a restricted cubic spline (RCS) function. If there was no evidence of non-linear association, we firstly estimated study-specific log HRs and 95% CIs for each 10 mg/day increment in soy isoflavone intake using the method introduced by Greenland and Longnecker [35]. Then we pooled study-specific log HRs to obtain a summarized effect size using a fixed effect model. Between-study heterogeneity was assessed by I^2^ statistic and the Egger test was used to detect publication bias.

Several sensitivity analyses were done to assess the robustness of dose–response meta-analysis: (1) dropping one of the studies included in the main dose–response meta-analysis each time; (2) dropping studies grouping participants according to soy frequency rather than amount of soy intake; (3) including only those studies which assessed soy intake in a more precise way (i.e. assessing both frequency and amount of soy intake using validated FFQs at baseline); or (4) including all the studies in the synthetic review, i.e. additionally including studies with extremely low level of soy isoflavone intake.

All statistical analyses were performed with Stata (version 15). All *P* values were two-sided and statistical significance was defined as *P* < 0.05.

## Result

### Patterns of soy consumption

Of the 300,852 female participants, 44.5% resided in urban areas, and the mean (SD) age at baseline was 50.9 (10.5) years (Table 1). At baseline, 12.0% reported never/rarely consuming soy foods (non-consumers) and 9.3% reported a regular consumption (i.e. ≥4 days/week). The estimated mean (SD) value of usual soy isoflavone intake was 9.4 (5.2) mg/day, corresponding to 7.5 (4.2) g/day of soybean equivalents. The spearman coefficient between baseline frequency group and usual amount quartile of soy intake was 0.76. Women consuming soy more frequently were more likely to live in urban areas, to have a higher level of education, and higher household income. They were also more likely to be taller, to have a family history of cancer, to be regular consumers of fresh fruit, red meat, fish and dairy products, and to have higher total energy intake. There were no obvious variation in age, BMI, level of physical activity and reproductive characteristics between soy consumption categories. Few of the women were ever smoker or weekly alcohol consumers.

**Table 1:** Baseline characteristics of participants by baseline frequency of soy consumption^a^

	Never or rarely (n = 36,009)	Monthly (n = 90,639)	1–3 days/week (n = 146,289)	≥ 4 days/week (n = 27,915)	Overall (n = 300,852)
Age in years, mean (SD)	51.7 (10.6)	51.1 (10.5)	50.6 (10.4)	51.2 (10.5)	50.9 (10.5)
Urban (%)	26.5	32.6	55.1	50.4	44.5
Education > 6 years (%)	36.6	39.8	45.8	52.7	43.3
Household income > 20,000 yuan/year (%)	31.2	37.2	42.3	47.9	40.7
Never smoker (%)	94.2	94.7	95.3	95.2	94.9
Weekly alcohol consumption (%)	2.0	2.0	2.1	2.5	2.1
Adult attained height in cm, mean (SD)	153.6 (6.1)	153.9 (6.0)	154.3 (5.9)	154.8 (5.8)	154.1 (6.0)
BMI in kg/m^2^, mean (SD)	23.7 (3.6)	23.7 (3.4)	23.9 (3.4)	23.9 (3.5)	23.8 (3.5)
Physical activity in MET-hour/day, mean (SD)	20.3 (12.0)	20.4 (12.5)	20.5 (12.9)	20.5 (13.4)	20.5 (12.8)
Age at menarche in years, mean (SD)	15.6 (2.1)	15.5 (2.0)	15.4 (1.9)	15.3 (1.9)	15.4 (2.0)
Parity, mean (SD)	2.3 (1.5)	2.3 (1.5)	2.2 (1.3)	2.1 (1.2)	2.2 (1.4)
Months of breast-feeding per child, mean (SD)	14.5 (9.0)	14.6 (8.1)	14.4 (7.0)	14.1 (6.8)	14.5 (7.8)
Post-menopause at baseline (%)	52.6	52.5	52.2	52.3	52.4
Ever use of oral contraceptives (%)	9.1	9.6	10.1	9.5	9.8
Family history of cancer (%)	16.3	16.5	16.6	17.7	16.6
Regular consumption of foods^b^					
Fresh fruit	23.7	25.6	34.5	44.9	31.7
Fresh vegetables	97.7	97.8	99.0	99.2	98.3
Preserved vegetables	24.9	21.8	22.6	25.4	22.8
Red meat	36.6	40.1	45.9	51.6	44.1
Poultry	0.7	0.7	0.9	2.4	1.0
Fish	6.2	7.9	8.5	12.9	8.5
Dairy	8.5	9.2	13.3	19.7	12.5
Total energy intake in kcal/day, median	1078.6	1139.3	1256.6	1280.1	1231.8

^a^Values for all variable except age, urban and total energy intake were standardized for age and region

^b^Participants eating foods for at least 4 days per week were regarded as regular consumers

### Association of soy consumption with incident breast cancer

During a mean follow-up of 10 years, 2289 women developed incident breast cancer. Among the 2,289 breast cancer cases, 1120 were identified among premenopausal women and 1169 among postmenopausal women at baseline. The mean (SD) age at breast cancer diagnosis was 56.6 (9.8) years. Table 2 shows the risk of incident breast cancer in relation to the frequency and usual amount of soy isoflavone intake. Overall, there was no evidence of any association between soy intake and breast cancer risk. Comparing with never/rare consumers, the multivariable-adjusted hazard ratios were 1.03 (95% CI 0.87–1.22) for women consuming soy foods 1–3 days per week and 0.98 (95% CI 0.80–1.22) for regular consumers. The trend tests were not statistically significant. Likewise, risk estimates were similar across quartiles of soy isoflavone intake in the total population.

**Table 2:** Association of soy consumption and risk of incident breast cancer: multivariable Cox models

	No. of cases	Person-years	Incidence rate (per 100,000 person-years)^b^	Adjusted hazard ratio (95% confidence intervals)		
				Model 1	Model 2	Model 3
*Baseline soy frequency*						
Never or rarely (Median 3.2 mg/day)	194	359,459	66.0	1.00	1.00	1.00
Monthly (Median 4.5 mg/day)	546	904,889	75.2	1.11 (0.94–1.31)	1.10 (0.93–1.31)	1.08 (0.91–1.28)
1-3 days/week (Median 14.4 mg/day)	1291	1,471,813	77.7	1.09 (0.92–1.28)	1.07 (0.91–1.27)	1.03 (0.87–1.22)
≥ 4 days/week (Median 19.1 mg/day)	258	279,390	78.7	1.05 (0.86–1.29)	1.03 (0.84–1.26)	0.98 (0.80–1.20)
*P* for trend^a^				0.817	0.998	0.537
*Quartiles of usual soy isoflavone intake*						
Q1 (Median 4.5 mg/day)	495	993,841	67.1	1.00	1.00	1.00
Q2 (Median 7.2 mg/day)	311	517,016	70.9	0.99 (0.84–1.17)	0.99 (0.83–1.17)	0.96 (0.81–1.13)
Q3 (Median 14.4 mg/day)	1,309	1,344,269	80.9	1.11 (0.97–1.28)	1.10 (0.96–1.26)	1.06 (0.92–1.22)
Q4 (Median 19.1 mg/day)	174	160,425	80.9	1.05 (0.86–1.28)	1.03 (0.84–1.26)	1.00 (0.81–1.22)
*P* for trend^a^				0.288	0.379	0.682

Model 1: Stratified by baseline age groups and study regions and adjusted for education attainment (no formal school, primary school, middle school, or high school or above) and household income (< 10000, 10000–19999, or ≥ 20000 yuan/year)

Model 2:Adjusted for variables in model 1 plus smoking status (never or ever), alcohol consumption (weekly alcohol consumer or non-weekly consumer), physical activity (0–10.9, 11.0–17.9, 18.0–29.4, or ≥ 29.5 MET-hour/day), baseline BMI (< 24.0, 24.0–27.9, or ≥ 28.0 kg/m^2^ according to overweight/obesity definition of Chinese population), and standing height (continuous)

Model 3: Adjusted for variables in model 2 plus age at menarche (< 10, 11, 12, 13, 14, 15, 16, 17, 18, or ≥ 19 years old), parity (0, 1, 2, or ≥ 3), average breastfeeding duration (0, 0.1–1, 1–2, or > 2 years), menopausal status and age at menopause (pre-menopausal, menopausal age < 45 years old, menopausal age 45–49 years old, menopausal age ≥ 50 years old), use of oral contraceptives (ever or never), family history of cancer (yes or no), consumption frequency of fresh fruit, fresh vegetables, preserved vegetables, red meat, poultry, fish and dairy products at baseline (never/rarely, monthly, 1–3 days/week, 4–6 days/week, or daily), and total energy intake

^a^Tests for trend were conducted by coding the groups as 1, 2, 3 and 4, respectively

^b^Values were adjusted for study region and age at study date

Baseline menopausal status did not appear to modify the results (Table 3, *P* = 0.856 for frequency-menopausal status interaction and *P* = 0.972 for quartile-menopausal status interaction). In the subgroup analysis according to BMI, higher soy intake seemed to be inversely associated the risk of breast cancer in women with BMI < 24 kg/m^2^, with a HR (95% CI) of 0.76 (0.60–0.96) for the highest soy frequency consumption group and a HR (95% CI) of 0.75 (0.54–1.04) for the highest quartile of soy consumption (Appendix Table 2). But no statistically significant interaction between soy intake and BMI was detected (Appendix Table 2: *P* = 0.131 for frequency-BMI interaction and *P* = 0.281 for quartile-BMI interaction; Appendix Tables 3 and 4). In sensitivity analyses, neither the exclusion of cases occurring during the first 2 years of follow-up, nor excluding those with a family history of cancer materially altered the results (data not shown).

**Table 3:** Soy intake and risk of incident breast cancer according to menopausal status

	No. of cases	Person-years	Incidence rate (per 100,000 person-years)^a^	Adjusted hazard ratio (95% confidence intervals)^b^
*Baseline frequency (P for interaction = 0.856)*				
Pre-menopause				
Monthly or less^**c**^	376	629,714	73.6	1.00
1–3 days/week	628	709,199	78.9	0.99 (0.85–1.15)
≥ 4 days/week	116	127,684	76.8	0.90 (0.71–1.13)
*P* for trend^d^				0.433
Post-menopause				
Monthly or less^**c**^	364	634,634	72.4	1.00
1–3 days/week	663	762,614	76.3	0.94 (0.81–1.09)
≥ 4 days/week	142	151,706	79.3	0.93 (0.75–1.15)
*P* for trend^d^				0.446
*Quartiles of usual soy isoflavone intake (P for interaction = 0.972)*				
Pre-menopause				
Q1	233	452,731	66.4	1.00
Q2	157	229,605	73.5	1.00 (0.79–1.28)
Q3	652	706,875	81.8	1.11 (0.91–1.35)
Q4	78	77,386	80.6	1.00 (0.74–1.35)
*P* for trend^e^				0.556
Post-menopause				
Q1	262	541,110	68.9	1.00
Q2	154	287,411	70.4	0.89 (0.70–1.13)
Q3	657	637,395	79.0	0.99 (0.81–1.21)
Q4	96	83,039	79.3	0.97 (0.73–1.28)
*P* for trend^e^				0.872

^a^Values were adjusted for study region and age at study date

^b^Adjusted for the same variables as model 3 in Table 2, except menopausal status

^**c**^The “Never or rarely” and “Monthly” groups were combined into the “Monthly or less” to ensure enough cases in each frequency category

^d^Test for trend was conducted by coding the “Monthly or less” group as 1, “1-3 days/week” group as 2, and “≥4 days/week” group as 3

^e^Test for trend was conducted by coding from the lowest to the highest quartile into 1, 2, 3 and 4, respectively

### Systematic review and dose–response meta-analysis

A total of 452 records were retrieved from Pubmed, Embase, Cochrane library and through hand searching. After removing duplicated records and abstract screening, 28 full-text articles were reviewed for eligibility checking (Appendix Figure 1). Of these, 11 studies were eligible with their characteristics and results of quality assessment presented in Appendix Tables 5 and 6.

In the dose–response meta-analysis, we further excluded three studies [15, 18, 20] because of markedly lower soy isoflavone intake when compared with other studies. The other eight studies were included in the present dose–response meta-analysis together with the CKB study, with a total of 631,498 women and 10,229 breast cancer cases. Seven [25, 26, 28, 30–32, 36] out of the eight studies used validated FFQs to assess soy intake while the FFQ in only one study [27] was not validated. Five studies [25, 26, 30–32] assessed both frequency and amount of soy food intake at baseline and calculated soy isoflavone intake based on the isoflavone content of different soy foods obtained from Food Composition Tables or published data. Two studies assessed soy food frequency at baseline and the portion size and isoflavone content were estimated from the validation study [28, 36]. One study collected information on soy food frequency and the amount of soy isoflavone intake was not available from either the baseline survey or the validation study [27]. Seven out of the eight individual studies included in the present dose–response meta-analysis adjusted for total energy intake when assessing the soy-breast cancer association [25, 26, 28, 30–32, 36]. Seven [25, 26, 28, 30–32, 36] out of the eight studies were of high and one study [27] of moderate quality according to the Newcastle–Ottawa quality assessment scale (NOS) scores (Appendix Table 6).

**Fig. 1:**
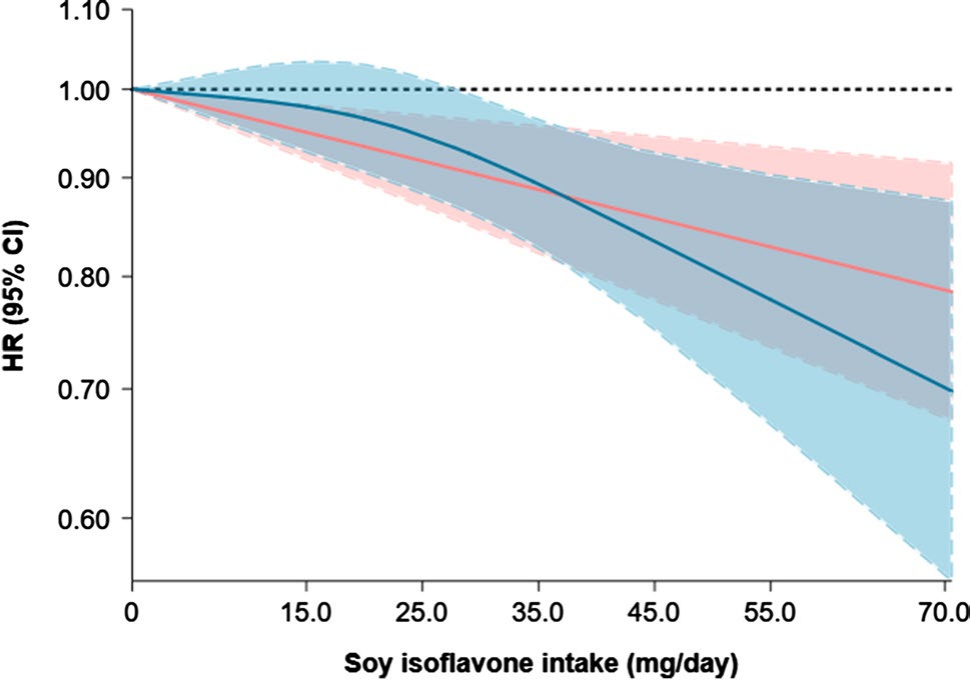
The relationship between incident breast cancer and soy isoflavone intake in dose–response meta-analysis of CKB and 8 published cohorts. Test for non-linear dose–response relation between soy isoflavone intake and incident breast cancer was statistically insignificant (*P* for non-linearity = 0.142). The blue line and shadow represent the hazard ratio (HR) and 95% confidence interval (CI) for the non-linear model while the red line (shadow) represents the HRs (95% CI) for the linear model

**Fig. 2:**
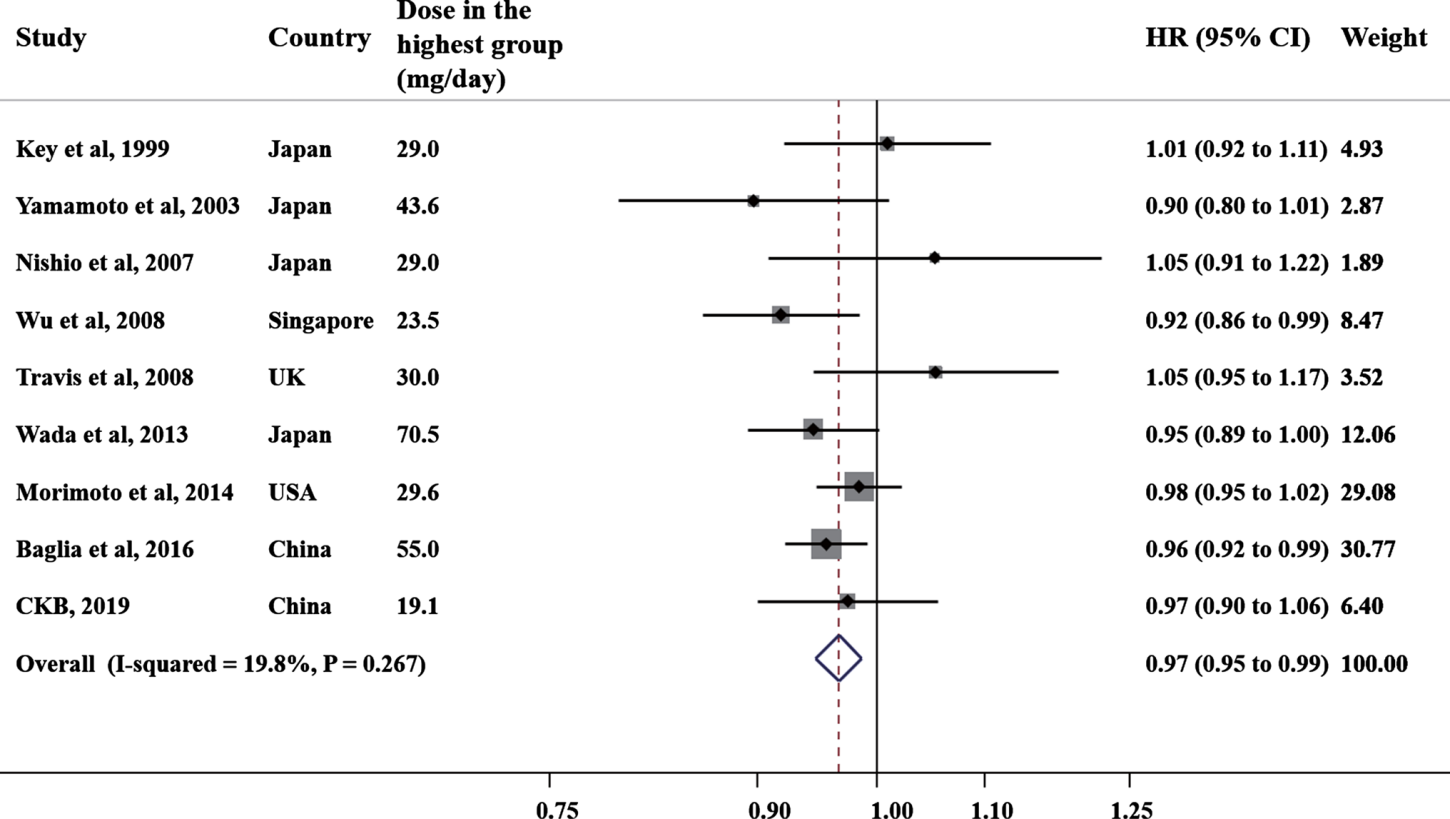
The forest plot of hazard ratio (HR) and 95% confidence interval (CI) of incident breast cancer for each 10 mg/day increment in soy isoflavone intake in dose–response meta-analysis of CKB and 8 published cohorts (fixed effect model). The sizes of square boxes are inversely proportional to the variances of logarithmic HRs

The dose–response meta-analysis using a RCS function failed to reject the linearity assumption between soy isoflavone intake and risk of breast cancer (*P* for non-linearity = 0.142; Fig. 1). Linear dose–response meta-analysis found that each 10 mg/day increment in soy isoflavone intake was associated with a mild reduced risk of breast cancer (HR: 0.97, 95% CI 0.95–0.99; Fig. 2). The between-study heterogeneity was low (I^2^ = 19.8%, *P* for heterogeneity = 0.267). The Egger test including the 9 studies in the dose–response meta-analysis found no publication bias (*P* = 0.606).

Sensitivity analyses by excluding one of the nine studies each time from the dose–response meta-analysis found that the risk estimates remained stable (Appendix Table 7). Sensitivity analysis by excluding two studies [27, 28] which reported HRs (95% CIs) according to soy frequency rather than soy intake amount revealed no major change in risk estimate (HR: 0.96, 95% CI 0.94–0.98; I^2^ = 22.1%, *P* for heterogeneity = 0.260). Sensitivity analysis by including the five studies [25, 26, 30–32] that assessed both frequency and amount of soy intake at baseline found similar risk estimate as the main dose–response meta-analysis (HR: 0.97, 95% CI 0.94–0.99; I^2^ = 35.9%, *P* for heterogeneity = 0.182). The dose–response meta-analysis of all 12 studies gave the same risk estimate (HR: 0.97, 95% CI 0.95–0.99; I^2^ = 0.0%, *P* for heterogeneity = 0.499) as the main dose–response meta-analysis of nine studies.

## Discussion

The CKB study, which enrolled over 300,000 women from 10 diverse regions from China, found no association between soy intake and incident breast cancer overall. The dose–response meta-analysis integrating the CKB study and other prospective studies from Asia and Western countries found that each 10 mg/day of soy isoflavone intake was associated with a 3% reduced breast cancer risk.

Findings from prospective studies on soy-breast cancer association were inconsistent, which may be due to different soy intake levels across different studies. Cohort studies conducted among women with low (< 5 mg/day in the highest consumption groups) [15, 18, 20] or moderate (20–30 mg/day in the highest consumption groups) [25–28] soy isoflavone intake found no clear association between soy and breast cancer risk. In the CKB study, the median amount of soy isoflavone intake in the highest consumption group was about 20 mg/day and no association was found between soy intake and breast cancer risk. This result was consistent with the four cohorts with moderate amount of soy intake [25–28]. In four studies in which the highest quartile or quintile intake groups had soy isoflavone > 40 mg/day [31, 32, 36] or the upper half intake group had a median intake at 23.5 mg/day (so upper quartile group is likely to have ~ 40 mg/day) [30]), reduced breast cancer risk was found for women among the highest soy consumption group compared to women among the lowest consumption group.

The soy intake level (mean: 7.5 g/day of soybean equivalents) was much lower among the CKB women than that assessed in the study conducted in Shanghai, China (median ~ 25 g/day of soybean equivalents) [32], which may be partially explained by the relatively lower level of soy intake among general Chinese adults than that among women in Shanghai. According to the three Chinese National Nutrition and Health Surveys conducted between 1992 and 2012, the mean daily intake of soy foods among Chinese adults was about 10–15 g of soybean equivalents, and remained stable throughout the past two decades [37]. The mean daily soy intake was slightly lower among CKB women compared with results from the three Chinese national nutrition and health surveys, while the average soy intake in women of the Shanghai study was much higher than general Chinese population.

We found an inverse association between higher soy food frequency and breast cancer among CKB women with lower BMI while no statistically significant association was observed among women with higher BMI. The Singapore Chinese study also reported different risks estimates among women with different BMI [30]. However, tests for multiplicative interaction between soy intake and BMI were statistically insignificant in both studies. Therefore, it is likely that different risk estimates for soy intake among women with different body sizes were due to chance.

The present dose–response meta-analysis observed a 3% (95% CI 1–5%) reduced risk of breast cancer for each 10 mg/day increment in soy isoflavone intake. Most of the cohort studies included in the present dose–response meta-analysis used validated FFQs to assessed soy intake. Most studies were of high quality according to the NOS scores which evaluated studies in terms of exposure and outcome measurement, confounding adjustment, follow-up duration and so on. Sensitivity analysis by excluding the study [27] which was of lower quality observed no change in the risk estimate, and sensitivity analysis by including exclusively the five studies that assessed both the frequency and amount of soy intake at baseline gave similar risk estimate, indicating the robustness of the present dose–response meta-analysis. Besides, between-study heterogeneity was low in the main dose–response meta-analysis as well as in sensitivity analyses, and the Egger test found no evidence of publication bias, indicating the reliability of the dose–response meta-analysis. In the present dose–response meta-analysis, the breast cancer risk reduced by each 10 mg/day of soy isoflavone intake as weakly as 3%, which may explain why most individual studies (including CKB) with low to moderate level of soy isoflavone intake failed to detect a statistically significant soy-breast cancer association.

According to the Dietary Guidelines for Chinese Residents in 2016, the recommended daily amount of soy food intake for adults were 15–25 g soybean equivalents [37]. The isoflavone content for 15–25 g/day soybean equivalents of different soy foods range between 10 and 50 mg/day (Appendix Table 8). Therefore, women consuming soy foods at the amount recommended by the Dietary Guidelines may be at 3–15% reduced risk of breast cancer according to the result of the present dose–response meta-analysis.

Could soy isoflavone supplement help? Two Western studies assessed soy supplement or soy isoflavone supplement, and neither of them found association of soy or soy isoflavone supplement with overall breast cancer risk [38, 39], though one study found that current soy isoflavone supplement was associated with reduced risk of estrogen receptor positive (ER+) breast cancer and increased risk of ER- breast cancer [39]. However, the dose of isoflavone in the supplements could vary in the study [39].

As the largest cohort study on soy-breast cancer association, the CKB study has several strengths, including large sample size, population from diverse areas across China, prospective cohort design, long duration of follow-up, unified method of exposure assessment across study regions, and stringent quality control of data. Our meta-analysis included only prospective cohort studies, which minimized recall bias.

There are several limitations, nevertheless. Firstly, the baseline questionnaire asked CKB participants about soy intake frequency rather than intake amount, and thus the usual amount of soy isoflavone was estimated by combing information from baseline surveys, two resurveys and the 24-HDRs. More large scale prospective studies using more precise exposure measurement methods are warranted to verify findings from the CKB study. Secondly, some energy-providing food items such as oil were not assessed in the CKB study, and therefore the calculated total energy was lower than the actual total energy intake. However, the risk estimates in the CKB study remained stable before and after adjustment for the calculated total energy intake. Thirdly, data on hormone receptor status of breast cancer were unavailable in the CKB study, so we were unable to assess the association of soy intake with breast cancer subtypes. Existing prospective evidence of soy-breast cancer subtype association has been scarce and of low statistical power due to small sample sizes [30, 32, 39]. Lastly, owing to the observational nature of studies included in the dose–response meta-analysis, we could not rule out the possibility of residual confounding caused by unmeasured factors. Large randomized controlled trials (RCT) may be desirable in causal inference but the feasibility of a large RCT on this issue is still a sticky issue.

## Conclusion

The CKB study demonstrated that moderate soy intake was not associated with breast cancer risk among Chinese women. Higher amount of soy intake might provide reasonable benefits for the prevention of incident breast caner. More large scale and well-designed prospective studies are needed to verify our findings.

## Electronic supplementary material

Below is the link to the electronic supplementary material.

Supplementary material 1 (DOCX 102 kb)
