# Supplementary material for: Soy intake and breast cancer risk: a prospective study of 300,000 Chinese women and a dose–response meta-analysis
Source: Eur J Epidemiol. 2019 Nov 21;35(6):567–78. doi: 10.1007/s10654-019-00585-4 (PMC7320952; doi:10.1007/s10654-019-00585-4)
Supplement: Supplementary file 1 — Supplementary material 1 (DOCX 102 kb) [file 10654_2019_585_MOESM1_ESM.docx]

**Appendix Material**

# Calculation of the usual amount of soy isoflavone intake

1. For each participant in the 2^nd^ resurvey, the daily amount of soy food (excluding soy milk) consumption was used to substituted the ***‘daily amount of soy consumption’*** since the consumption of soymilk was negligible.
2. The daily amount for participants in the baseline survey were computed using data collected during the 2^nd^ resurvey. Firstly, participants in the baseline were stratified by age (i.e. <40, 40 to 59, or ≥60 years), study regions (i.e. Harbin, Qingdao, Gansu, Henan, Haikou, Suzhou, Liuzhou, Sichuan, Zhejiang, or Hunan), and baseline soy consumption groups (i.e. ‘never/rarely’, ‘monthly’, ‘1-3 days per week’, or ‘≥4 days per week’). Secondly, the median value of ***‘daily amount of soy consumption’*** for participants in the 2^nd^ resurvey in each stratum was used as a proxy of ***‘baseline daily amount of soy consumption’* (variable B)** for baseline participants in the corresponding stratum***.*** This process was based on the assumption that the daily amount of soy consumption did not vary much from the baseline to the 2^nd^ resurvey (i.e. people may change their frequency of soy foods consumption but the daily amount remains roughly the same).
3. The mean number of soy consumption days (**variable D_n_**) assigned to each consumption group was 0 days for the ‘never/rarely’ group (D_1_=0), 2.5 days for the ‘monthly’ group (D_2_=2.5), 8.6 days for the ‘1-3 days per week’ group (D_3_=8.6), and 24 days for the ‘≥ 4 days per week’ group (D_4_=24).
4. The information from the 1^st^ resurvey (**Table S1**) was used to assess the variation in soy frequency from the baseline to 1^st^ resurvey. The ‘usual amount of soy consumption per day’ for participants at n^th^ baseline group (**Variable U_n_**) was estimated using the following formula:

**U_n_** =**B** ×（**D_n_ ×F_ni_**）.

F_ni_ is the percentage of participants at the n^th^ baseline frequency group switching to the i^th^ frequency group in the 1^st^ resurvey. For example, for participants at the ‘never/rarely’ group in baseline survey, 34.0% remained at the ‘never/rarely’ group in 1^st^ resurvey, 42.3% switched to the ` monthly’ group, 21.3% switched to the `‘1-3 days per week’ group, and 2.4% switched to the ‘≥ 4 days per week’ group.

Therefore, the ‘usual amount of soy consumption per day’ for participants at ‘never/rarely’ group in baseline survey equal to **B** × (0 × 34.0% + 2.5 × 42.3% + 8.6 × 21.3% + 24 ×2.4%). The ‘usual amount of soy consumption per day’ for participants at other baseline group (i.e. ‘monthly’, ‘1-3 days per week’, or ‘≥4 days per week’) were calculated using the same approach.

1. According to the 24-HDRs, there were more than 10 kinds of soy foods consumed by CKB participants and the proportion that the **j^th^** kind of soy food amount taking up the total soy food amount (**Variable** **P_j_**) was estimated. The proportions that different soy foods contributing to total soy food amount were 44.5% for fresh tofu, 16.0% for soybeans (mature seeds), 10.6 % for pressed tofu, 7.7% for sprouted soybeans, 6.3% for edamame, 5.3% for soymilk skin or film, 3.9% for fried tofu, 2.5% for soybean flakes, 1.8% for sufu, and ~1.5% for other soy foods.
2. The ‘usual amount of soy isoflavone consumption per day’ was calculated according to the following formulas:

$$\sum_{j=1}^{23} (\boldsymbol{‘usual amount of soy consumption per day’} \times\boldsymbol{Pj} \times\boldsymbol{IFj})$$

**IF_j_** was the isoflavone content of the j^th^ kind of soy food according to the Chinese Food Composition Tables[1, 2].

1. CKB participants were categorized into 4 groups according to the quartiles of usual soy isoflavone intake. The agreement between baseline soy consumption frequency and quartiles of usual amount was assessed by means of spearman correlation coefficient.

# Appendix Table 1. Variation in soy frequency from baseline survey to first resurvey based on 11,936 participants attending the first resurvey in 2008

| **Baseline frequency groups** | | **First resurvey frequency groups** | | | |
| --- | --- | --- | --- | --- | --- |
|  |  | **n (F_ni_ / %)^a^** | | | |
|  |  | **Never/rarely** | **Monthly** | **1-3days/week** | **≥4 days/week** |
|  |  | **1** | **2** | **3** | **4** |
| ^1^ | Never/rarely | 533 (34.0) | 664 (42.3) | 334 (21.3) | 38 (2.4) |
| ^2^ | Monthly | 734 (20.3) | 1,501 (41.5) | 1,241 (34.3) | 144 (4.0) |
| ^3^ | 1-3days/week | 543 (9.6) | 1,319 (23.2) | 3,262 (57.4) | 562 (9.9) |
| ^4^ | ≥4 days/week | 46 (4.3) | 175 (16.5) | 618 (58.3) | 222 (20.9) |

^a^ F_ni_ is the percentage of participants at the n^th^ baseline frequency group switching to the i^th^ frequency group in the 1^st^ resurvey.

**Appendix Table 2. Soy intake and risk of incident breast cancer according to body mass index (BMI)**

|  | **No. of cases** | **Person-years** | **Incidence rate (per 100,000 person-years)**^a^ | **Adjusted hazard ratio (95% confidence intervals)**^b^ |
| --- | --- | --- | --- | --- |
| **Baseline frequency (*P* for interaction=0.131)** | | | | |
| BMI< 24.0 kg/m^2^ |  |  |  |  |
| Monthly or less^c^ | 348 | 698,371 | 62.6 | 1.00 |
| 1-3 days/week | 603 | 799,059 | 65.7 | 0.93 (0.80-1.08) |
| ≥ 4 days/week | 105 | 153,841 | 57.8 | 0.76 (0.60-0.96) |
| *P* for trend^d^ |  |  |  | 0.033 |
| BMI≥ 24.0 kg/m^2^ |  |  |  |  |
| Monthly or less^c^ | 392 | 565,977 | 85.6 | 1.00 |
| 1-3 days/week | 688 | 672,754 | 91.5 | 1.00 (0.87-1.16) |
| ≥ 4 days/week | 153 | 125,549 | 103.2 | 1.09 (0.88-1.34) |
| *P* for trend^d^ |  |  |  | 0.508 |
| **Quartiles of usual soy isoflavone intake (*P* for interaction=0.281)** | | | | |
| BMI< 24.0 kg/m^2^ |  |  |  |  |
| Q1 | 237 | 562,649 | 58.8 | 1.00 |
| Q2 | 173 | 311,014 | 66.4 | 0.96 (0.76-1.21) |
| Q3 | 580 | 691,952 | 66.3 | 0.96 (0.77-1.20) |
| Q4 | 66 | 85,656 | 56.7 | 0.75 (0.54-1.04) |
| *P* for trend^e^ |  |  |  | 0.209 |
| BMI≥ 24.0 kg/m^2^ |  |  |  |  |
| Q1 | 258 | 431,192 | 78.3 | 1.00 |
| Q2 | 138 | 206,001 | 75.7 | 0.93 (0.72-1.19) |
| Q3 | 729 | 652,317 | 97.5 | 1.14 (0.95-1.38) |
| Q4 | 108 | 74,769 | 108.8 | 1.24 (0.95-1.62) |
| *P* for trend^e^ |  |  |  | 0.086 |

^a^ Values were adjusted for study region and age at study date.

^b^ Adjusted for the same variables as model 3 in table 2, except body mass index.

^c^ The “Never or rarely” and “Monthly” groups were combined into the “Monthly or less” to ensure enough cases in each frequency category.

^d^ Test for trend was conducted by coding the “Monthly or less”, “1-3 days/week”, and “≥4 days/week” group as 1, 2 and 3, respectively

^e^ Test for trend was conducted by coding from the lowest to the highest quartile into 1, 2, 3 and 4, respectively.

# Appendix Table 3. Soy intake and breast cancer risk according to body mass index (BMI) among pre-menopausal women

|  | **No. of cases** | **Person-years** | **Incidence rate (per 100,000 person-years)^a^** | **Adjusted hazard ratio (95% confidence intervals)**^b^ |
| --- | --- | --- | --- | --- |
| **Baseline frequency (*P* for interaction=0.811)** | | | | |
| BMI< 24.0 kg/m^2^ |  |  |  |  |
| Monthly or less^c^ | 195 | 356,977 | 68.7 | 1.00 |
| 1-3 days/week | 334 | 412,616 | 71.5 | 0.92 (0.75-1.13) |
| ≥ 4 days/week | 62 | 76,876 | 67.9 | 0.79 (0.58-1.09) |
| *P* for trend§ |  |  |  | 0.162 |
| BMI≥ 24.0 kg/m^2^ |  |  |  |  |
| Monthly or less^c^ | 181 | 272,736 | 80.2 | 1.00 |
| 1-3 days/week | 294 | 296,583 | 88.9 | 1.07 (0.86-1.32) |
| ≥ 4 days/week | 54 | 50,808 | 89.8 | 1.03 (0.74-1.44) |
| *P* for trend^d^ |  |  |  | 0.714 |
| **Usual amount quartile (*P* for interaction=0.547)** | | | | |
| BMI< 24.0 kg/m^2^ |  |  |  |  |
| Q1 | 121 | 261,831 | 60.7 | 1.00 |
| Q2 | 94 | 139,192 | 70.7 | 0.99 (0.72-1.36) |
| Q3 | 337 | 397,525 | 74.9 | 1.03 (0.76-1.38) |
| Q4 | 39 | 47,921 | 65.6 | 0.80 (0.52-1.23) |
| *P* for trend^e^ |  |  |  | 0.579 |
| BMI≥ 24.0 kg/m^2^ |  |  |  |  |
| Q1 | 112 | 190,900 | 73.5 | 1.00 |
| Q2 | 63 | 90,413 | 76.0 | 1.00 (0.69-1.45) |
| Q3 | 315 | 309,350 | 91.7 | 1.18 (0.90-1.54) |
| Q4 | 39 | 29,465 | 102.9 | 1.27 (0.84-1.93) |
| *P* for trend^e^ |  |  |  | 0.180 |

**^a^** Values were adjusted for study region and age at study date.

^b^ Adjusted for the same variables as model 3 in table 2, except for BMI and menopausal status.

^c^ The “Never or rarely” and “Monthly” groups were combined into the “Monthly or less” to ensure enough cases in each frequency category.

^d^ Test for trend was conducted by coding the three groups as 1, 2 and 3, respectively

^e^ Test for trend was conducted by coding from the lowest to the highest quartile into 1, 2, 3 and 4, respectively.

# Appendix Table 4. Soy intake and breast cancer risk according to body mass index (BMI) among post-menopausal women

|  | **No. of cases** | **Person-years** | **Incidence rate (per 100,000 person-years)**^a^ | **Adjusted hazard ratio (95% confidence intervals)**^b^ |
| --- | --- | --- | --- | --- |
| **Baseline frequency (*P* for interaction=0.087)** | | | | |
| BMI< 24.0 kg/m^2^ |  |  |  |  |
| Monthly or less^c^ | 153 | 341,393 | 56.8 | 1.00 |
| 1-3 days/week | 269 | 386,444 | 59.7 | 0.93 (0.74-1.18) |
| ≥ 4 days/week | 43 | 76,965 | 47.4 | 0.71 (0.49-1.03) |
| *P* for trend^d^ |  |  |  | 0.101 |
| BMI≥ 24.0 kg/m^2^ |  |  |  |  |
| Monthly or less^c^ | 211 | 293,241 | 90.2 | 1.00 |
| 1-3 days/week | 394 | 376,170 | 93.8 | 0.96 (0.79-1.16) |
| ≥ 4 days/week | 99 | 74,741 | 112.1 | 1.10 (0.84-1.43) |
| *P* for trend^d^ |  |  |  | 0.623 |
| **Usual amount quartile (*P* for interaction=0.587)** | | | | |
| BMI< 24.0 kg/m^2^ |  |  |  |  |
| Q1 | 116 | 300,818 | 60.7 | 1.00 |
| Q2 | 79 | 171,822 | 70.7 | 0.91 (0.65-1.29) |
| Q3 | 243 | 294,427 | 74.9 | 0.88 (0.63-1.22) |
| Q4 | 27 | 37,735 | 65.6 | 0.67 (0.41-1.11) |
| *P* for trend^e^ |  |  |  | 0.173 |
| BMI≥ 24.0 kg/m^2^ |  |  |  |  |
| Q1 | 146 | 240,292 | 73.5 | 1.00 |
| Q2 | 75 | 115,589 | 76.0 | 0.88 (0.63-1.23) |
| Q3 | 414 | 342,967 | 91.7 | 1.10 (0.85-1.42) |
| Q4 | 69 | 45,304 | 102.9 | 1.20 (0.85-1.70) |
| *P* for trend^e^ |  |  |  | 0.302 |

^a^ Values were adjusted for study region and age at study date.

^b^ Adjusted for the same variables as model 3 in table 2, except body mass index and menopausal status.

^c^ The “Never or rarely” and “Monthly” groups were combined into the “Monthly or less” to ensure enough cases in each frequency category.

^d^Test for trend was conducted by coding the three groups as 1, 2 and 3, respectively

^e^ Test for trend was conducted by coding from the lowest to the highest quartile into 1, 2, 3 and 4, respectively.

**Methods of systematic literature review and dose-response meta-analysis.**

Using the following searching strategies, we searched on PubMed, Embase and Cochrane library from their dates of inception to March 2019 for all possible articles published in English.

**1. PubMed:** ("Breast Neoplasms"[Mesh] OR "breast cancer"[Text Word] OR "breast carcinoma"[Text Word] OR "breast neoplasm"[Text Word] OR "breast tumor"[Text Word]) AND ("Soybeans"[Mesh] OR "Soy Foods"[Mesh] OR "Isoflavones"[Mesh] OR "soy"[Text Word] OR "soybean"[Text Word] OR "soya"[Text Word] OR "soyfood"[Text Word] OR "tofu"[Text Word] OR "miso"[Text Word] OR "soymilk"[Text Word] OR "fermented bean curd"[Text Word] OR "isoflavone"[Text Word] OR "daidzein"[Text Word] OR "genistein"[Text Word]) AND ("Cohort Studies"[Mesh] OR "prospective"[Text Word] OR "longitudinal"[Text Word] OR "follow-up"[Text Word] OR "cohort "[Text Word])

**2. Embase:** ('breast neoplasms'/exp OR 'breast neoplasms' OR 'breast cancer'/exp OR 'breast cancer' OR 'breast carcinoma'/exp OR 'breast carcinoma' OR 'breast neoplasm' OR 'breast tumor'/exp OR 'breast tumor') AND ('soybeans'/exp OR 'soybeans' OR 'soy foods'/exp OR 'soy foods' OR 'isoflavones'/exp OR 'isoflavones' OR 'soy' OR 'soybean'/exp OR 'soybean' OR 'soya'/exp OR 'soya' OR 'soyfood' OR 'tofu'/exp OR 'tofu' OR 'miso' OR 'soymilk'/exp OR 'soymilk' OR 'fermented bean curd' OR 'daidzein'/exp OR 'daidzein' OR 'genistein'/exp OR 'genistein' OR 'isoflavone'/exp OR 'isoflavone') AND ('cohort studies'/exp OR 'cohort' OR 'prospective' OR 'longitudinal'/exp OR 'longitudinal' OR ' follow-up')

**3. Cochrane library:** (mh "Breast Neoplasms") or (breast cancer or breast carcinoma or breast neoplasm or breast tumor) in Title Abstract Keyword AND (mh "Soybeans") or (mh "Soy Foods") or (mh "Isoflavones") or “soy” or “soybean” or “soya” or “soyfood” or “tofu” or “miso” or “soymilk” or “fermented bean curd” or “isoflavone” or “daidzein” or “genistein” in Title Abstract Keyword AND (mh " cohort studies) or (cohort or prospective or longitudinal or longitudinal or follow-up)

Two authors (YW and MG) conducted data extraction independently using standard extraction form. Data items extracted were: publication year, name of first author, cohort name, study area, baseline age of participants, sample size, follow-up duration, covariates adjusted in the most fully-adjusted model, and number of cases, person years, mean or median value of soy isoflavone intake, the most fully-adjusted HR and corresponding 95%CI for each consumption category. For the CKB study, the corresponding data items for four groups of soy isoflavone intake were extracted, too. When person years were not presented directly in the publications, we obtained the values through personal communication or through multiplying the mean follow-up time by number of participants in each category. If the mean or median values of soy isoflavone intake were not presented directly in original articles, several ways were tried to estimate them: 1) the median values were obtained directly through personal communication; 2) the midpoint of the lower and upper boundaries of corresponding category was used as a proxy value[3]; 3) the median value of the highest category was set at 1.5 times the lower boundary if the highest category was open ended[3]; 4) for studies reporting the mean or median intake of a single kind of soy isoflavone rather than total soy isoflavones, amount of total soy isoflavone was estimated according to the proportion the kind of soy isoflavone taking up the total soy isoflavones in soy foods[4]; and 5) for studies categorizing participants using soy frequencies rather than intake amount of soy or soy isoflavone, the median amount of soy isoflavone intake was estimated by multiplying intake frequency per day with average portion sizes.

The quality of individual cohort studies included in the present review was assessed using the Newcastle-Ottawa quality assessment scale (NOS). The NOS scale was used for quality assessment of non-randomized studies in meta-analysis and its content validity and inter-rater reliability has been established[5]. In this scale, studies were assessed according to eight items involving assessment of selection, comparability and outcome, with a maximum score of 9 points. We regarded scores of 0-3, 4-6, and 7-9 as low, moderate, and high quality, respectively[6].

17 articles excluded:

5 Study populations were reported in other articles

5 The exposure was not soy (or soy isoflavone), or the outcome was not incident breast cancer

3 Reviews or editorial

2 The amount of soy isoflavone intake was unavailable

1 The relative risks were unavailable

1 Retrospective cohort study

Full-text articles assessed

for eligibility
(n = 28)

CKB study

Studies included in qualitative synthesis
(n =12)

**Appendix Figure 1. Flow diagram of record search and study selection process**

Records excluded
(n =279)

Studies included in quantitative synthesis (dose-response meta-analysis)
(n = 9)

Records identified through hand searching (n=16)
(n =132 )

Records identified in Cochrane library
(n =32)

Records identified in Embase database
(n =263 )

Records identified in Pubmed database
(n =141 )

Records screened
(n =307)

Duplicates removed
(n =145)

## Identification

## Eligibility

## Included

## Screening

# Appendix Table 5. Prospective cohort studies on the association of soy intake with breast cancer

| **Study** | **Country** | **Sample size** | **Breast cancer case** | **Age at baseline (years)** | **Follow-up years** | **Categories of exposure** | **Covariates in fully adjusted model** |
| --- | --- | --- | --- | --- | --- | --- | --- |
| Key  1999[7] | Japan | 34,759 | 427 | NA | mean 14.1 | **Tofu intake frequency (isoflavone: mg/day^a^)**  1 time/week (8.0)  2–4 times/week (20.0)  5 times/week (29.0) | age, calendar period, city, age at time of bombing, and dose of radiation |
| Horn-Ross  2002[8] | USA | 111,526 | 711 | 21-103 | mean 2 | **Isoflavone intake (mg/day)**  Quintile 1 (0.32)  Quintile 2 (0.88)  Quintile 3 (1.36)  Quintile 4 (1.84)  Quintile 5 (3.12) | age, race, daily caloric intake, family history of breast cancer, age at menarche, nulliparity/age at first full-term pregnancy, physical activity, and an interaction term for BMI and menopausal status |
| Yamamoto  2003[9] | Japan | 21,852 | 179 | 40-59 | mean 9.6 | **Soy isoflavone (mean**^b^**, mg/day)**  Quartile 1 (11.9)  Quartile 2 (22.4)  Quartile 3 (34.5)  Quartile 4 (43.6) | area; age; age at menarche; number of pregnancies; menopausal status; age at first pregnancy; active and passive smoking; alcohol consumption; leisure time physical activities; educational level; total energy; and meat, fish, vegetable, and fruit consumption. |
| Touillaud  2006[10] | France | 26,868 | 402 | NA | mean 4.2 | **Soy isoflavone (mean, mg/day)**  Quartile 1 (0.011)  Quartile 2 (0.025)  Quartile 3 (0.032)  Quartile 4 (0.074) | Age as the time scale, and adjusted for education, height, BMI, age at menarche, personal history of benign breast disease or lobular carcinoma in situ (yes or no), family history of breast cancer, use of oral contraceptive, age at first full-term pregnancy and parity, area, alcohol, and energy |
| Nishio  2007[11] | Japan | 30,454 | 145 | 40-79 | mean 7.6 | **Tofu intake frequency (isoflavone: mg/day**^c^**)**  < 3 times/week (16.0)  3-4 times/week (20.0)  Almost daily (29.0) | age, study area, family history of breast cancer, age at menopause, age at first birth, parity, use of exogenous female hormone, smoking, consumption of green leafy vegetables, walking time, BMI, and total energy intake |
| Wu  2008[12] | Singapore | 34,028 | 629 | 45-74 | mean 9.9 | **Soy isoflavone (median, mg/day**^d^**)**  Group 1 (7.67)  Group 2 (23.51) | age, years of interview, dialect, education, family history of breast cancer, parity, age when period became regular, menopausal status, BMI, and n-3 fatty acid |
| Travis  2008[13] | UK | 37,643 | 585 | 20-90 | median 7.4 | **Soy isoflavone (mean, mg/day)**  Group 1 (5^e^)  Group 2 (15^e^)  Group 3 (30^f^) | age, height, BMI, age at menarche, age at first birth, parity, alcohol consumption, daily energy intake, menopausal status, and current hormone replacement therapy use |
| Hedelin  2008[14] | Sweden | 45,448 | 1,014 | 30-49 | mean 13 | **Total isoflavonoid**  **(median, mg/day**^g^**)**  Quartile 1 (0.0035)  Quartile 2 (0.007)  Quartile 3 (1)  Quartile 4 (2) | age, energy, BMI, oral contraceptives, age at first pregnancy, age at menarche, parity, breast cancer among sisters or mothers, smoking, education, and selected food groups and nutrient densities |
| Wada  2013[15] | Japan | 15,607 | 172 | 35+ | mean 14 | **Soy isoflavone intake (mg/day)**  Quartile 1 (18.6)  Quartile 2 (32.8)  Quartile 3 (44.0)  Quartile 4 (70.6) | age, BMI , physical activity score, smoking status, alcohol consumption, years of education, age at menarche, age at first delivery, menopausal status at the baseline, parity, history of hormone replacement therapy, and total energy |
| Morimoto  2014[16] | USA | 84,550 | 4769 | 45-75 | mean 12.5 | **Soy isoflavone (median, mg/day)**  Quartile 1 (1.7)  Quartile 2 (4.8)  Quartile 3 (9.1)  Lower quartile 4 (16.0)  Upper quartile 4 (29.6) | ethnicity, age, BMI, age at menarche, age at first live birth, parity, menopausal status, education, oral contraceptive use, menopausal hormone use, family history of breast cancer, total energy intake, alcohol consumption, smoking, diabetes, and hypertension |
| Baglia  2016[17] | China | 70,578 | 1034 | 40-70 | median 13.2 | **Soy isoflavone (mg/day)**  Quintile 1 (11.1)  Quintile 2 (19.9)  Quintile 3 (27.8)  Quintile 4 (37.5)  Quintile 5 (55.0) | age, BMI, age at first live birth, physical activity, education, family history of breast cancer, season of recruitment, menopause, and total energy intake |

**^a^** Portion sizes were not available in this study and therefore portion sizes were estimated from another cohort study conducted in Japan[11].

^b^ The mean value of total isoflavone consumption was estimated according to the proportion of genistein (58%) taking up the total isoflavones in soy foods[4].

**^c^** The mean isoflavone intake for each tofu frequency was estimated by combing soy food frequencies at baseline with portion sizes and soy isoflavone content of different soy foods from the 12-day dietary records[11].

^d^ The median intake of isoflavone was obtained through personal communication.

^e^ The mean value of each category was the midpoint of the lower and upper boundaries of corresponding quartile[3].

^f^ The midpoint of the highest category was set at 1.5 times the lower boundary[3].

^g^ The mean value of each category was estimated from the median and range of isoflavone intake of the cohort without incident breast cancer.

# Appendix Table 6. The Newcastle-Ottawa quality assessment scale score of studies included in the qualitative analysis

| **Study** | **Representativeness of the exposed cohort** | **Selection of the non-exposed cohort** | | **Exposure assessment**^a^ | **No breast cancer at baseline** | **Controlling for confounders**^b^ | **Outcome assessment (medical records or disease registers)** | **Adequate follow-up time (>5 years)** | **Follow-up rate (>80%)** | **Overall score** | |
| --- | --- | --- | --- | --- | --- | --- | --- | --- | --- | --- | --- |
| Key, 1999 | 0 | | 1 | 0 | 1 | 0 | 1 | 1 | 1 | 5 |  |
| Horn-Ross, 2002 | 0 | | 1 | 1 | 1 | 2 | 1 | 0 | 1 | 7 |  |
| Yamamoto, 2003 | 1 | | 1 | 1 | 1 | 1 | 1 | 1 | 1 | 8 |  |
| Touillaud, 2006 | 0 | | 1 | 1 | 1 | 2 | 1 | 0 | 1 | 7 |  |
| Nishio, 2007 | 1 | | 1 | 0 | 1 | 2 | 1 | 1 | 1 | 8 |  |
| Wu, 2008 | 1 | | 1 | 1 | 1 | 2 | 1 | 1 | 1 | 9 |  |
| Travis, 2008 | 1 | | 1 | 1 | 1 | 2 | 1 | 1 | 1 | 9 |  |
| Hedelin, 2008 | 1 | | 1 | 1 | 1 | 2 | 1 | 1 | 1 | 9 |  |
| Wada, 2013 | 1 | | 1 | 1 | 1 | 2 | 1 | 1 | 1 | 9 |  |
| Morimoto, 2014 | 1 | | 1 | 1 | 1 | 2 | 1 | 1 | 1 | 9 |  |
| Baglia, 2016 | 1 | | 1 | 1 | 1 | 2 | 1 | 1 | 1 | 9 |  |
| CKB, 2019 | 1 | | 1 | 1 | 1 | 2 | 1 | 1 | 1 | 9 |  |

^a^ Using a validated FFQ and amount of soy isoflavone intake for each consumption level was reported in the original article.

^b^ Controlling for age, BMI, reproductive factors and total energy intake.

# Appendix Table 7. Dose-response meta-analyses by excluding one of the nine studies each time

| **Excluding study** | **Pooled HR (95%CI) for each 10 mg/day increment in isoflavone intake** | **I^2^ (%)** | **P value for heterogeneity** |
| --- | --- | --- | --- |
| Key et al, 1999 | 0.97 (0.95-0.99) | 22.9 | 0.247 |
| Yamamoto et al, 2003 | 0.97 (0.95-0.99) | 16.8 | 0.297 |
| Nishio et al, 2007 | 0.97 (0.95-0.99) | 19.3 | 0.277 |
| Wu et al, 2008 | 0.97 (0.95-0.99) | 9.4 | 0.357 |
| Travis et al, 2008 | 0.96 (0.94-0.98) | 6.1 | 0.383 |
| Wada et al, 2013 | 0.97 (0.95-0.99) | 25.0 | 0.230 |
| Morimoto et al, 2014 | 0.96 (0.94-0.98) | 19.7 | 0.274 |
| Baglia et al, 2016 | 0.98 (0.95-1.00) | 26.2 | 0.220 |
| CKB, 2019 | 0.97 (0.95-0.99) | 29.5 | 0.192 |

# Appendix Table 8. The process of calculating the isoflavone content for 15~25 g soybean equivalents of different soy foods^a^

|  | **A. protein content of soy foods (g/100g soy foods )** | **B. coefficients to convert soybean equivalents into amount of different soy foods** | **C. soy foods (g) of 15g soybean equivalents^c^** | **D. soy foods (g) of 25 g soybean equivalents^c^** | **E: isoflavone content of soy foods (mg/100g soy foods )** | **F. isoflavone content (mg) for 15 g soybean equivalents of different soy foods** | **G. isoflavone content (mg) for 25 g soybean equivalents of different soy foods** |
| --- | --- | --- | --- | --- | --- | --- | --- |
|  |  | =35/A | =15**×**B | =25**×**B |  | =C**×**E/100 | =D**×**E/100 |
| **Tofu** | 6.6 | 5.3 | 79.5 | 132.6 | 30.0 | 23.8 | 39.7 |
| **Tofu, pressed, raw** | 14.9 | 2.3 | 35.2 | 58.7 | 33.9 | 11.9 | 19.9 |
| **Soybeans, green, raw (includes edamame)** | 13.1 | 2.7 | 40.1 | 66.8 | 49.0 | 19.6 | 32.7 |
| **Soybeans, mature seeds, raw (China)** | 35.0 | 1.0^b^ | 15.0 | 25.0 | 118.3 | 17.7 | 29.6 |
| **Soybean, sprouted** | 4.5 | 7.8 | 116.7 | 194.4 | 12.5 | 14.6 | 24.3 |
| **Tofu, fried** | 17 | 2.1 | 30.9 | 51.5 | 34.8 | 10.7 | 17.9 |
| **Soybean, curd, fermented** | 10.9 | 3.2 | 48.2 | 80.3 | 34.7 | 16.7 | 27.8 |
| **Soymilk skin or film** | 44.6 | 0.8 | 11.8 | 19.6 | 196.1 | 23.1 | 38.5 |
| **Soybean, flakes** | 24.5 | 1.4 | 21.4 | 35.7 | 62.3 | 13.4 | 22.3 |
| **Soymilk powder** | 19.7 | 1.8 | 26.6 | 44.4 | 109.5 | 29.2 | 48.6 |

^a^ According to the Dietary Guidelines for Chinese Residents in 2016, the recommended daily amount of soy food intake for adults were 15~25 g soybean equivalents[18].

^b^ The soy protein content of soybeans was 35 g/100g and it was considered as the reference.

^c^ The conversion coefficients were based on the protein content of different soy foods and the soybeans[2, 18].

**References**

1. Yang Y, Wang G, Pan X. Chinese Food Composition Tables 2009. Beijing, China Beijing University Medical Press; 2009.

2. Yang Y. Chinese Food Composition Tables 2018. sixth ed. Beijing, China: Beijing University Medical Press; 2018.

3. Rong Y, Chen L, Zhu T, Song Y, Yu M, Shan Z et al. Egg consumption and risk of coronary heart disease and stroke: dose-response meta-analysis of prospective cohort studies. BMJ (Clinical research ed). 2013;346:e8539. doi:10.1136/bmj.e8539.

4. Zaheer K, Humayoun Akhtar M. An updated review of dietary isoflavones: nutrition, processing, bioavailability and impacts on human health. Critical reviews in food science and nutrition. 2017;57(6):1280-93. doi:10.1080/10408398.2014.989958.

5. Wells G, Shea B, O'Connell D, Peterson J, Welch V, Losos M et al. The Newcastle-Ottawa Scale (NOS) for assessing the quality of nonrandomized studies in meta-analyses. 2011. http://www.ohri.ca/programs/clinical_epidemiology/oxford.asp.

6. Zheng JS, Hu XJ, Zhao YM, Yang J, Li D. Intake of fish and marine n-3 polyunsaturated fatty acids and risk of breast cancer: meta-analysis of data from 21 independent prospective cohort studies. BMJ (Clinical research ed). 2013;346:f3706. doi:10.1136/bmj.f3706.

7. Key TJ, Sharp GB, Appleby PN, Beral V, Goodman MT, Soda M et al. Soya foods and breast cancer risk: a prospective study in Hiroshima and Nagasaki, Japan. Br J Cancer. 1999;81(7):1248-56. doi:10.1038/sj.bjc.6690837.

8. Horn-Ross PL, Hoggatt KJ, West DW, Krone MR, Stewart SL, Anton H et al. Recent diet and breast cancer risk: the California Teachers Study (USA). Cancer Causes Control. 2002;13(5):407-15.

9. Yamamoto S, Sobue T, Kobayashi M, Sasaki S, Tsugane S. Soy, isoflavones, and breast cancer risk in Japan. J Natl Cancer I. 2003;95(12):906-13. doi:DOI 10.1093/jnci/95.12.906.

10. Touillaud MS, Thiebaut AC, Niravong M, Boutron-Ruault MC, Clavel-Chapelon F. No association between dietary phytoestrogens and risk of premenopausal breast cancer in a French cohort study. Cancer epidemiology, biomarkers & prevention : a publication of the American Association for Cancer Research, cosponsored by the American Society of Preventive Oncology. 2006;15(12):2574-6. doi:10.1158/1055-9965.EPI-06-0543.

11. Nishio K, Niwa Y, Toyoshima H, Tamakoshi K, Kondo T, Yatsuya H et al. Consumption of soy foods and the risk of breast cancer: findings from the Japan Collaborative Cohort (JACC) Study. Cancer Causes Control. 2007;18(8):801-8. doi:10.1007/s10552-007-9023-7.

12. Wu AH, Koh WP, Wang R, Lee HP, Yu MC. Soy intake and breast cancer risk in Singapore Chinese Health Study. Br J Cancer. 2008;99(1):196-200. doi:10.1038/sj.bjc.6604448.

13. Travis RC, Allen NE, Appleby PN, Spencer EA, Roddam AW, Key TJ. A prospective study of vegetarianism and isoflavone intake in relation to breast cancer risk in British women. Int J Cancer. 2008;122(3):705-10. doi:10.1002/ijc.23141.

14. Hedelin M, Lof M, Olsson M, Adlercreutz H, Sandin S, Weiderpass E. Dietary phytoestrogens are not associated with risk of overall breast cancer but diets rich in coumestrol are inversely associated with risk of estrogen receptor and progesterone receptor negative breast tumors in Swedish women. J Nutr. 2008;138(5):938-45.

15. Wada K, Nakamura K, Tamai Y, Tsuji M, Kawachi T, Hori A et al. Soy isoflavone intake and breast cancer risk in Japan: from the Takayama study. Int J Cancer. 2013;133(4):952-60. doi:10.1002/ijc.28088.

16. Morimoto Y, Maskarinec G, Park SY, Ettienne R, Matsuno RK, Long C et al. Dietary isoflavone intake is not statistically significantly associated with breast cancer risk in the Multiethnic Cohort. Br J Nutr. 2014;112(6):976-83. doi:10.1017/S0007114514001780.

17. Baglia ML, Zheng W, Li H, Yang G, Gao J, Gao YT et al. The association of soy food consumption with the risk of subtype of breast cancers defined by hormone receptor and HER2 status. Int J Cancer. 2016;139(4):742-8. doi:10.1002/ijc.30117.

18. The Chinese Nutrition Society. The Dietary Guidelines for Chinese Residents (2016). Beijing, China: People's Medical Publishing House; 2016.
